# Identification of a subunit of NADH-dehydrogenase as a p49/STRAP-binding protein

**DOI:** 10.1186/1471-2121-9-8

**Published:** 2008-01-29

**Authors:** Xiaomin Zhang, Gohar Azhar, Scott Helms, Ying Zhong, Jeanne Y Wei

**Affiliations:** 1 From the Donald W. Reynolds Department of Geriatrics, The University of Arkansas for Medical Sciences and Geriatric Research, Education, and Clinical Center, Little Rock, AR, USA; 2 Central Arkansas Veterans Healthcare System, Geriatric and Extended Care, Little Rock, AR, USA; 3 Central Arkansas Veterans Healthcare System, Little Rock, AR, USA

## Abstract

**Background:**

The p49/STRAP (or SRFBP1) protein was recently identified in our laboratory as a cofactor of serum response factor that contributes to the regulation of SRF target genes in the heart.

**Results:**

In the present study, we report that NDUFAB1, a nuclear encoded subunit of NADH dehydrogenase, represented the majority of the cDNA clones that interacted with p49/STRAP in multiple screenings using the yeast two-hybrid system. The p49/STRAP and NDUFAB1 proteins interacted and co-localized with each other in the cell. The p49/STRAP protein contains four classic nuclear localization sequence motifs, and it was observed to be present predominantly in the nucleus. Overexpression of p49/STRAP altered the intracellular level of NAD, and reduced the NAD/NADH ratio. Overexpression of p49/STRAP also induced the deacetylation of serum response factor.

**Conclusion:**

These data suggest that p49/STRAP plays a role in the regulation of intracellular processes such as cardiac cellular metabolism, gene expression, and possibly aging.

## Background

A novel protein, p49/STRAP, was recently identified in our laboratory [1]. The initial observations indicated that p49/STRAP is a cofactor of serum response factor (SRF) contributing to the regulation of SRF target genes in the heart. This gene was later named by The Human Genome Organization (HUGO) as SRFBP1 based on our initial finding that p49/STRAP binds to SRF. The p49/STRAP mRNA and protein are expressed in multiple tissues, including heart, brain, liver, skeletal muscle and kidney. An increase of p49/STRAP expression has been observed in the myocardium of human and mouse with advancing age, and also in transgenic mice suffering from cardiomyopathy [1,2].

Our previous studies suggested that the expression of a number of cardiac genes are regulated by a protein complex containing SRF and it's multiple binding proteins, including p49/STRAP and Zipzap/p200 [1-3]. We had noticed that the protein complexes obtained by immunoprecipitation assays with either p49/STRAP or SRF antibody contained more components than we had anticipated, suggesting that additional proteins may be present in the complex [1-3]. The p49/STRAP protein interacts directly with the SRF transcriptional activation domain. Therefore, it is possible that a protein complex formed around this domain or those proteins that bind to p49/STRAP may have a significant effect on SRF activation. In an attempt to determine the identity of these putative proteins, we used p49/STRAP protein fragments as bait in the yeast two-hybrid system. We found that NDUFAB1, a subunit of NADH dehydrogenase (a nuclear encoded subunit of mitochondrial Complex I), represented the majority of cDNA clones interacting with p49/STRAP in the multiple screenings. Overexpression of p49 changed the intracellular NAD/NADH ratio and reduced the amount of acetylated-SRF protein. Therefore, p49 may play a role in the process of protein acetylation and deacetylation, thereby modulating transcriptional regulation.

## Methods

### Yeast Two-hybrid Screening and Bioinformatic Analysis

Yeast two-hybrid screening was performed as previously described [1]. Briefly, one "bait" construct containing the N-terminus (residues 1–256) and another one containing the C-terminus (residues 250–427) of the p49/STRAP protein were used to screen cDNA libraries generated from a human heart and Hela cells (Clontech). A total of three separate screenings was performed. Positive target cDNA clones were subjected to a series of analyses according to the manufacturer's instruction manual. The final positive clones were sequenced and their sequences were compared with the Genbank nucleotide database.

### Bioinformatics Analysis

The p49/STRAP and NDUFAB1 sequences were subjected to analyses using web-based and/or freely-downloaded bioinformatic tools, including "PSORT II" [4], "iPSORT" [5], "WoLF PSORT", "BaCelLo" [6], "pTARGET" [7], "CELLO version 2" [8], "Golgi Localization Predictor" [9], "HSLPred" [10], "GoCore" and "SignalP" [11].

### Cloning of Full-length Coding Region Sequence of NDUFAB1 gene

The majority of the cDNA clones interacting with the N-terminal bait of the p49/STRAP protein in the yeast two-hybrid screening matched a single gene in the GenBank database, NDUFAB1, which is a subunit of NADH dehydrogenase (OMIM 25010; EC 1.6.5.3). The full-length coding region of the human NDUFAB1 gene was amplified by PCR using a human heart cDNA sample (Clontech) and then subcloned into the expression vectors.

### Plasmid Constructs

A pcDNA3-Flag-NDUFAB1 construct was generated by ligating the NDUFAB1 coding region in frame with Flag into the pcDNA3-Flag vector. A pcDNA3-mCherry-NDUFAB1 construct was generated by ligating the mCherry red fluorescent protein (RFP, a gift from Dr. Roger Tsien, University of California, San Diego) in frame with NDUFAB1 into the pcDNA3 vector. The p49/STRAP-EGFP fusion protein construct and the pcDNA3-HA-p49/STRAP construct were generated as previously reported [1]. All the plasmid constructs were subjected to sequencing analysis to verify the open reading frame sequence.

### Generation of Recombinant Adenoviruses

The p49/STRAP recombinant adenovirus was generated using the AdEasy system, a generous gift from Dr. Vogelstein (The Johns Hopkins Oncology Center, Baltimore) [12]. The generation, purification and titration of the recombinant adenoviruses Ad-EGFP and Ad-p49/STRAP were performed according to the protocol described by He et al. [12].

### Tissue Culture and Transfection Assays

The H9C2 cells, NIH3T3 cells and neonatal rat cardiac myocytes were cultured as previously described [1,13,14]. Transient transfection was carried out using the desired expression plasmid constructs and the Lipofectamine 2000 reagent (Invitrogen). At approximately 4 hr after the transfection was initiated, cells were placed in DMEM medium containing 10% newborn bovine serum and incubated at 37°C overnight.

### Co-immunoprecipitation and Western Blotting

The expression plasmid constructs containing p49/STRAP and NDUFAB1 were cotransfected into NIH3T3 cells by using Lipofectamine 2000 (Invitrogen) as previously described [1]. At 48 h after the transfection, cells were harvested, and the whole-cell lysate was isolated. The co-immunoprecipitation and Western blotting were carried out as previously described [1]. Antibodies that were employed included monoclonal antibodies HA.11 (Covance), FLAG (M2, Sigma), SRF (Abcam) and an anti-acetyl-Lysine antibody (clone 4G12, Upsate); polyclonal antibodies HA (Santa Cruz), Flag (Sigma), a SRF antibody which recognizes the C-terminus of SRF protein (Santa Cruz) and p49/Strap [1].

### Fluorescence Microscopy

The expression plasmids "EGFP-p49/STRAP" and "mCherry-NDUFAB1" were used for the transfection. At approximately 24, 48, and 72 hours after transfection, the expression of EGFP-p49/STRAP and mCherry-NDUFAB1 were examined by fluorescence microscopy using a Nikon Eclipse E600 equipped with CoolSNAP-ES digital camera and MetaVue 6.2 software.

The intensity of fluorescence was measured using the "ImageJ 1.37" program (National Institutes of Health, Bethesda, MD). Background noise was subtracted. The area of, and total fluorescence signal from, nuclear and non-nuclear cellular regions were measured separately. The cytoplasm superior and inferior to the nucleus was estimated to have a p49/STRAP concentration similar to that in the perinuclear region. It was further estimated that the cytoplasmic overlay on the nucleus was thinner than that of the surrounding region. For these reasons, the total fluorescence in the nuclear region was estimated to consist of p49 in the nucleus proper, and an amount of p49 represented by the product of the intensity of signal in the extranuclear region and the area of the nucleus itself.

Nuclear p49 signal = Total Nuclear Signal - (Extranuclear Signal Intensity × Nuclear Area)

In addition, the cytosolic fraction of p49 was estimated to be a ratio of cytosolic signal to the total signal.

$$\text{Cytosolic p49 (\%)}=\frac{\text{100 }\times \text{ Cytosolic Signal}}{(\text{Cytosolic }+\text{ Nuclear})\text{ Signal}}$$

### NAD/NADH Assay

The H9C2 cells were used for the assessment of NAD/NADH ratio in response to p49/STRAP gene expression. The NAD assay was performed using a protocol modified from the methods described by Zerez et al. [15] as well as Bernofsky and Swan [16], in which H9C2 cells were infected with 25 MOI of recombinant adenoviruses 48 hours before the assay was performed. The values are expressed as means ± S.D. A p value of less than 0.05 was considered to be significant.

## Results

### NDUFAB1 is a p49/STRAP binding protein

p49/STRAP was first identified as a transcription cofactor of SRF [1]. To explore a broad spectrum of potential p49/STRAP binding proteins, we employed a yeast two-hybrid system in which two overlapping cDNA fragments covering the entire p49 protein were used as baits to screen two human cDNA libraries. The utilization of separate bait fragments, p49/STRAP N-terminus (p49-N) and p49/STRAP C-terminus (p49-C), was intended to maximize the screening efficiency and to distinguish the binding proteins that may selectively interact with the different parts of the p49 protein.

A human heart cDNA library derived from terminally differentiated cardiomyocytes and non-cardiomyocyte cells, and a Hela cell cDNA library generated from highly proliferating tumor cells were selected to represent the cells with different biological features.

When the human heart cDNA library was screened with p49-N bait, 42 out of 44 (95%) of the final target clones that were analyzed matched a single gene, NDUFAB1, which is a subunit of NADH dehydrogenase I. When the Hela cell cDNA library was screened, 8 out of 20 (40%) clones matched NDUFAB1 (table 1). Since NDUFAB1 cDNA clones were the main p49/STRAP interacting protein in two screenings with p49-N bait, we wondered whether NDUFAB1 could also be a major target for the p49-C bait screening.

**Table 1:** The NDUFAB1 protein selectively binds to the N-terminus of p49 protein (p49-N).

**Origin of cDNA Library**	**Screening bait**	**Clones positive for NDUFAB1**	**Main target**
Haert	p49-N	95%	NDUFAB1
Hela	p49-N	40%	NDUFAB1
Hela	p49-C	0%	Other

Three separate yeast two-hybrid screenings were performed using p49-N and p49-C as bait, respectively. When p49-N was used to screen human heart cDNA library, 42 out of 44 (95%) of the positive clones that interacted with p49-N were NDUFAB1. When p49-N was used to screen Hela cell cDNA library, 8 out of 20 (40%) of the positive clones were NDUFAB1. However, when p49-C was used to screen Hela cell cDNA library, none of the clones was NDUFAB1. These results indicate that p49-N selectively binds to NDUFAB1.

**Figure 1:**
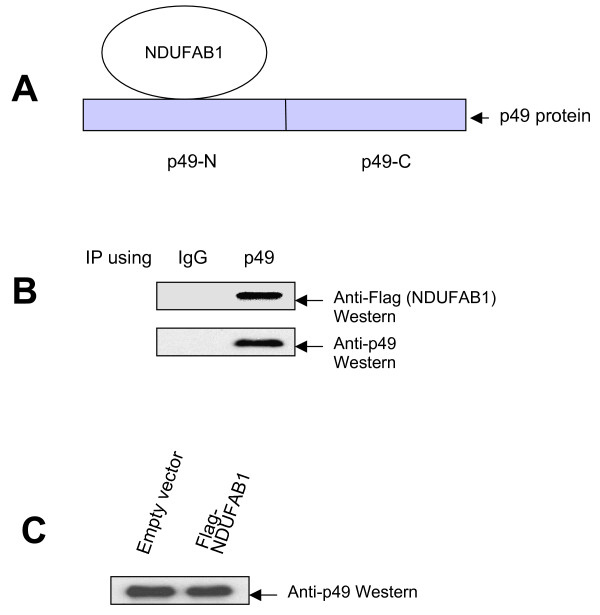
**NDUFAB1 binds to p49/STRAP**. **A**. Schematic representation indicating that NDUFAB1 binds to the N-terminus of p49/STRAP protein. **B**. H9C2 cells were transfected with Flag-NDUFAB1 plasmid construct and harvested 24 hours later. An anti-IgG antibody (used as a control) and an anti-p49 antibody were used in the co-immunoprecipitation assay. The results show that p49 antibody co-immunoprecipitated the endogenous p49 protein and the Flag-NDUFAB1 protein, indicating that Flag-NDUFAB1 was bound to endogenous p49 protein. **C**. Flag-NDUFAB1 expression did not change p49 protein level in the cells (figure 1c).

In a third screening, the p49-C bait plasmid was used to screen the Hela cell library and 41 final target clones were analyzed. However, no clone matched to NDUFAB1 (0%) (table 1). These data indicate that NDUFAB1 protein selectively binds the N-terminus of p49/STRAP (figure 1a).

To confirm the interaction between p49/STRAP and NDUFAB1 in the mammalian cells, we performed immunoprecipitation assays. As shown in figure 1b, immunoprecipitation of the endogenous p49/STRAP protein with anti-p49 antibody co-precipitated the NDUFAB1 protein. The expression of NDUFAB1 plasmid construct did not change the level of p49/STRAP protein in the cells (figure 1c).

### Bioinformatics analysis of p49/STRAPand NDUFAB1

To determine the functional and conserved domains in p49/STRAP and NDUFAB1, we used web-based bioinformatic tools to analyze the protein sequences of these two genes.

**Figure 2:**
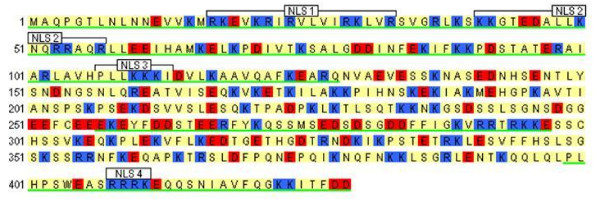
**Bioinformatics analysis of p49 sequence**. Bioinformatics analysis of the sequence of human p49 protein shows that there are four classic nuclear localizing sequences (NLS's), three of them are in the N-terminal region. NLS1 & NLS2 are bipartite nuclear localization sequences. NLS3 has a pattern-7 motif, and NLS4 has a pattern-4 motif. Blue (residue K or R) indicates positively charged (basic) residues, red (residue D or E) indicates negatively charged (acidic) residues. The sections underlined in green are highly conserved across human, rat, and mouse.

Analysis of p49/STRAP showed that it contains as many as four classic NLS motifs (figure 2). Two bipartite NLS motifs were located at the N-terminus of the sequence, including NLS #1 at codons 17–33, and NLS #2 at codons 41–57. NLS #3 is a classic pattern-7 (pat7) NLS motif with seven amino acid residues (PLLKKKI) at 107–113, and NLS #4 is a classic pattern-4 NLS motif with four residues (RRRK) at 408–411. Overall, the intracellular localization of p49/STRAP was predicted to be 65.2% nuclear, 21.7% cytoplasmic, 4.3% mitochondrial, 4.3% in vesicles of secretory system and 4.3% in endoplasmic reticulum (table 2).

**Table 2:** Bioinformatics estimate of intracellular localization probability of p49 and NDUFAB1.

**Intracellular site**	**p49/STRAP**	**NDUFAB1**
Nucleus	65.2%	8.7%
Cytoplasm	21.7%	8.7%
Vesicles of secretory	4.3%	0.0%
Mitochondria	4.3%	78.3%
Endoplasmic reticulum	4.3%	0.0%
Cytoskeleton	0.0%	4.3%

Multiple bioinformatics programs were used to analyze the potential intracellular localization of the p49 and NDUFAB1 proteins (see details in "Methods"). The results estimate that p49 protein is primarily localized within the nucleus, and NDUFAB1 is primarily localized in the mitochondria, although both proteins have the potential to be in other intracellular compartments as well.

Analysis of NDUFAB1 revealed that NDUFAB1 was estimated to be 78.3% mitochondrial, 8.7% nuclear, 8.7% cytoplasmic and 4.3% cytoskeletal (table 2).

### Intracellular localization of NDUFAB1

**Figure 3:**
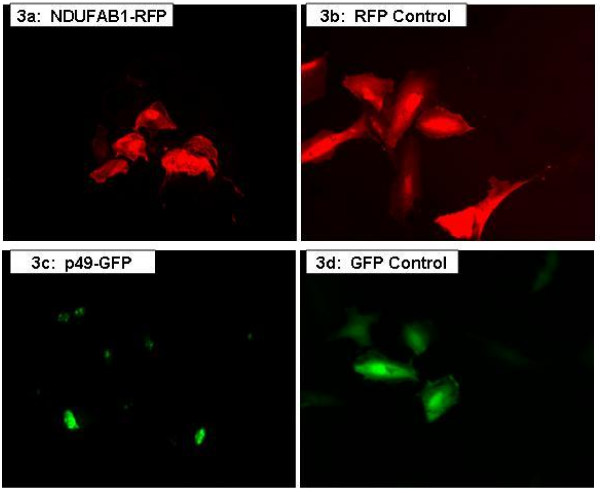
**Intracellular localization of p49 and NDUFAB1**. Fluorescent images of H9C2 cells transfected with NDUFAB1-RFP expression plasmids (**a**, top left) and RFP control (**b**, top right), demonstrate both nuclear and cytoplasmic distribution. The p49-GFP fusion protein appears to be primarily localized within the nucleus, especially the nucleoli (**c**, bottom left). The GFP-only control protein is distributed evenly throughout the cells (**d**, bottom right). GFP = green fluorescent protein; RFP red fluorescent protein.

NDUFAB1, which is a nuclear-encoded protein, is a subunit of the mitochondrial respiratory Complex I. To visualize the localization of NDUFAB1 in living cells, a red fluorescent protein (RFP), mCherry, was linked in-frame with NDUFAB1 and expressed in the H9C2 cells. As shown in figure 3a, the RFP-NDUFAB1 was observed to be present in both the cytoplasm and nucleus.

### Intracellular localization of p49/STRAP

**Figure 4:**
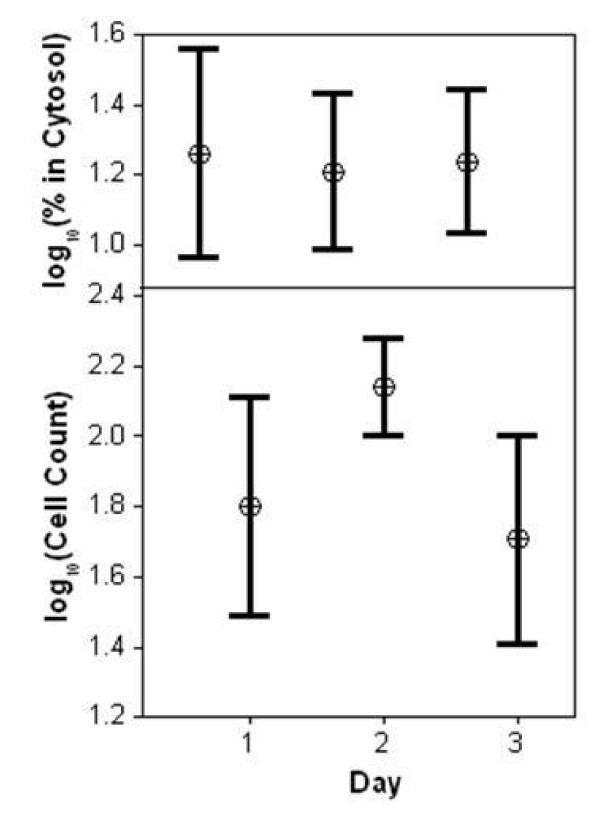
**Intracellular localization of p49 protein in cultured H9C2 cells**. Image analyses was performed on more than 200 H9C2 cells transfected with p49-GFP at 1,2 and 3 days after transfection. Cytosolic and nuclear signal intensity of p49 was computed using "Image J" program (see description in "Methods"). Top Panel: No significant relationship was found between p49-GFP cytosolic fraction and day post-transfection(Day, x axis). Bottom Panel: Images of cells with p49-GFP evident in the extranuclear region were measured after subtracting the background noise. The images were grouped based on the number of days post-transfection. In our studies, cell growth displayed a negative quadratic function during days 1–3 post-transfection (R2adj = 0.273, regression not shown).

The EGFP-p49/STRAP expression plasmid was transfected into H9C2 cells, neonatal cardiac myocytes and NIH3T3 cells. In the un-stimulated H9C2 cells, EGFP-p49/STRAP fusion protein was observed predominantly within the nucleus (figures 3c &4a). Nevertheless, in a small subset of the H9C2 cells, p49/STRAP was also observed in both nucleus and cytoplasm. Similar results were also observed in neonatal cardiac myocytes and NIH3T3 cells (data not shown).

**Figure 5:**
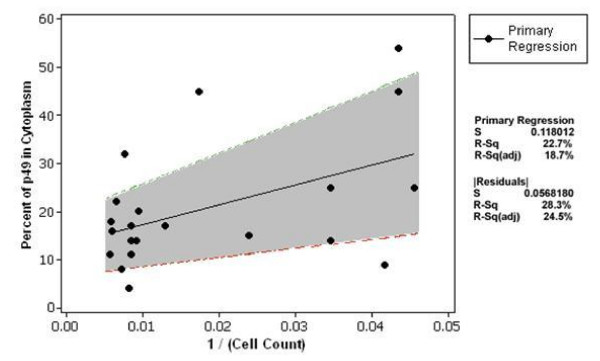
**Statistical indication that only a minority of the p49 protein is observed in the cytoplasm**. Based on our analyses (see detail in the "Methods" section), approximately less than 30% of the protein is located in the cytoplasm. Cells were counted using brightfield microscopy and the images were grouped by days post-transfection as shown in figure 4. It is interesting to note that both the absolute value of, and variability in, the cytosolic percent seem to be reduced with increased confluence in cell count. The absolute residuals were regressed against the cell count, and the resulting function added (+/-) to the function of the original regression, resulting in the "band" shown in gray. Taken together, the simple least squares regression and residual regression on cell count account for >43% of the variability in cytosolic percent. This implies that p49 is localized predominantly in the nucleus. The result is independent of day of culture, and only slightly influenced by degree of cell confluence.

To determine whether the incubation time after transfection could affect the localization of the p49 protein, cells were also observed at three time points (24 hours, 48 hours and 72 hours) after transfection. At all three time points, p49 localized predominantly in the nucleus in the majority of the cells, with no appreciable relationship with the time post-transfection (figures 4 and 5). However, the cytosolic localization of total p49/STRAP did seem to be slightly related to the degree of confluency (figure 5).

**Figure 6:**
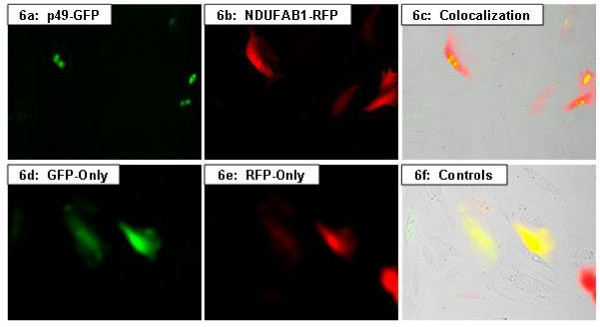
**Co-expression of p49 and NDUFAB1**. Fluorescent images of H9C2 cells showing uneven distribution p49-EGFP fusion protein alone, in green (**a**) and NDUFAB1-RFP fusion protein alone, in red (**b**). Co-expression of p49 and NDUFAB1 fusion proteins indicates co-localization of these two proteins that appear as yellow in the nuclear region (**c**). These panels, taken together with those of **figs. 3a-3d**, suggest a more focal localization of p49-GFP compared to control (**a, c**) in these cells. However, for the NDUFAB1-RFP construct (**b, c**), the distribution usually appears to be more even throughout the cell, similar to that of GFP-only and RFP-only controls (**d, e, f**). GFP = green fluorescent protein; RFP = red fluorescent protein.

When H9C2 cells were transfected with both p49/STRAP and NDUFAB1 expression plasmids, co-localization of the p49/STRAP and NDUFAB1 proteins was observed (figure 6).

### p49/STRAP expression affected NAD/NADH ratio

**Figure 7:**
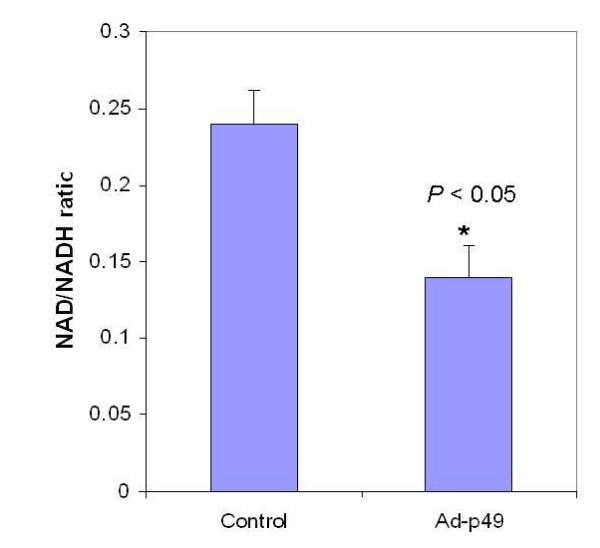
**Overexpression of p49/STRAP changes the intracellular NAD/NADH ratio**. H9C2 cells were infected with either recombinant adenovirus containing empty vector (control) or p49/STRAP gene (Ad-p49), respectively. After 48 hours, the cells were harvested and the cell lysate samples were subjected to NAD and NADH assay(see methods). The results indicate that p49/STRAP overexpression reduces the intracellular NAD/NADH ratio by approximately 40% in H9C2 cells (*p *< 0.05, n = 4).

NDUFAB1 is a subunit of NADH dehydrogenase that regulates intracellular NAD, NADH concentrations and the NAD/NADH ratio. To determine whether the interaction between p49/STRAP and NDUFAB1 would have any effect on NADH dehydrogenase activity, thereby affecting the NAD/NADH ratio, p49/STRAP was delivered into cells with recombinant adenovirus, and the cycling assay for NAD/NADH was then performed [15,16]. The results revealed that NAD concentration was 258 ± 12.4 nM in control adenovirus treated cells, but 165 ± 15.0 nM in p49/STRAP adenovirus treated cells; NADH concentration was 1065 + 15.5 nM in control adenovirus treated cells, but 1105 ± 24.6 nM in p49/STRAP adenovirus treated cells. The NAD/NADH ratio was 0.24 ± 0.016 in control adenovirus treated cells, while 0.14 ± 0.012 in p49/STRAP adenovirus treated cells. The results indicate that p49/STRAP adenovirus reduced the intracellular NAD/NADH ratio by approximately 40% (*p *< 0.05, n = 4) (figure 7).

**Figure 8:**
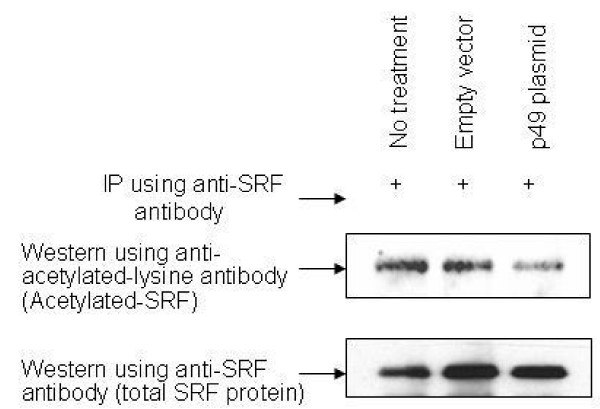
**p49/STRAP reduces the level of acetylated-SRF protein**. H9C2 cells were transfected with p49 plasmid construct (p49-plasmid) or with empty vector (empty vector) and harvested after 24 hours. H9C2 cells without transfection (No treatment) were used as controls. Equal amounts of the protein from the above samples were used to immunoprecipitate the SRF protein, using an antibody recognizing the C-terminal SRF protein (Santa Cruz). The immunoprecipitated SRF proteins were then subjected to SDS-PAGE and Western blotting. The level of total SRF protein in the samples was determined using the same anti-SRF antibody. The acetylated-SRF was detected by an antibody specifically recognizing the acetyl-lysine (clone 4G12, Upstate biotechnology). The results indicate that p49/STRAP overexpression reduces the level of acetylated-SRF protein.

Overexpression of p49/STRAP also reduced the acetylation of SRF (figure 8). These data implicate p49 as an inducer of SRF deacetylation.

## Discussion

The p49/STRAP protein was initially isolated by our group as a transcription cofactor of SRF, which modulates the transcriptional regulation of SRF-target genes [1]. In the present study, we found that p49/STRAP contained four classic NLS motifs. The p49 protein predominantly localized within the nucleus, but was also present at low levels in the cytoplasm. In addition, we found that p49/STRAP interacted with NDUFAB1, a subunit of the mitochondrial Complex I which regulates the redox status of NAD/NADH, and showed that NDUFAB1 could be observed in both the cytoplasm and nucleus. Furthermore, we found that overexpression of p49/STRAP altered the intracellular level of NAD and NADH, and reduced the NAD/NADH ratio. Overexpression of p49/STRAP also resulted in reduction of the acetylation of SRF. These results suggest that via interacting with a component of Complex I, p49/STRAP plays a role in the regulation of NAD and NADH which influence fundamental cellular processes such as cellular metabolism, gene expression, ion channel regulation and possibly aging [17-19].

It is known that Complex I, an enzyme complex that is located on the inner mitochondrial membrane catalyzes the transfer of electrons from NADH to coenzyme Q (CoQ). In the redox process, the complex translocates electrons across the inner membrane, helping to build the electrochemical potential used to produce ATP [20]. However, the exact catalytic mechanism remains incompletely established. Complex I is the largest respiratory complex, which contains 46 separate subunits. Of the 46 subunits, seven are encoded by the mitochondrial genome, while the rest are encoded by the nuclear genome.

NDUFAB1 is one of the nuclear-encoded subunits of Complex I, which contains a phosphopantetheine attachment site (DLGLDSLDQVEIIMAM), Characteristic of acyl carrier proteins. NDUFAB1 also contains an EF-hand calcium binding domain (DIDAEKLMCPQEI) [21]. Deletion of NDUFAB1 homolog in N. crassa results in impaired assembly of Complex I and a four-fold increase in lysophospholipid content of mitochondrial membranes, suggesting that NDUFAB1 is required for the assembly of Complex I, and that it is also involved in fatty acid synthesis [22]. Although NDUFAB1 was considered to be a membrane bound component of Complex I, recent evidence indicates that soluble NDUFAB1 protein has been found in the mitochondria, suggesting that NDUFAB1 may have other functions [23].

In the present study, NDUFAB1 was identified as a major binding protein to the p49/STRAP-N region. NDUFAB1 accounted for 95% of the cDNA clones derived from human heart and 40% of the cDNA clones derived from human Hela cells, which interacted with the p49-N region. NDUFAB1 mRNA is expressed in a wide range of human tissues. The highest expression levels are observed in adult heart, skeletal muscle and fetal heart [21]. Although majority of the cDNA clones derived from human heart and Hela cells interacted with the p49-N region, none of the clones interacted with the p49-C region. These data suggest that the high binding rate of NDUFAB1 to p49/STRAP N-terminus is unlikely to be random, and is unlikely to be associated merely with the expression level of NDUFAB1 in the tissue origin of the cDNA library. Rather, this suggests that the NDUFAB1 protein binds selectively to the N-terminus, but not the C-terminus, of the p49/STRAP protein.

The translocation of a protein from the cytoplasm through the nuclear pore complex and into the nucleoplasm is usually initiated through the interaction between an NLS motif on the cargo protein with the NLS motif binding site on importin-α [24]. The classic nuclear localization sequence generally conform to one of three types, known as pattern-4 (pat4), pattern-7 (pat7) (both are monopartite) and bipartite motifs [25-27]. The "pat4" NLS motif consists of a continuous stretch of four basic amino acids (lysine or arginine) or three basic amino acids associated with histidine or proline. The "pat7" NLS motif starts with a proline and is followed within three residues by an amino acid sequence containing three basic residues out of four. The third type of NLS motif, known as "bipartite" motif, consists of two basic amino acids, a 10 amino acid spacer and a five amino acid sequence containing at least three basic residues [25-27]. All four NLS motifs of the p49/STRAP protein comply with the classic definitions of NLS motifs. Our bioinformatic analyses also suggest that p49/STRAP could potentially localize in the other cellular compartments; likewise, NDUFAB1 may also be in the nucleus.

It is well documented that a single gene can give rise to more than one protein products with different sizes and with the potential to be localized to different intercellular compartments. These proteins can be derived from alternative splicing, or differential usage of translational initiation signals, thereby containing or lacking specific compartment targeting signals.

Recent studies indicate that even a single protein product can have the potential for dual intracellular localization. For example, wild-type p53 has been observed in cytoplasm and nucleus in both normal and tumor cells [28]. p53 protein is accumulated in mitochondria and nuclei of cardiac myocytes after treatment with Adriamycin [29]. Chronological observation of p53 protein revealed that upon TPA treatment, p53 was first translocated into mitochondria, and then into nucleus [30]. Other examples of protein with dual localization include BCL2, SRF, ERK, Myopodin, 14-3-3 proteins, MKP-1 protein, thioredoxin and BRCA1 [31-38].

It is considered that the localization signal sequence(s) in proteins play a major role in compartment localization of the proteins. Proteins that harbor two separate signals or an overlapping ambiguous signal, and even only one signal sequence may follow dual distribution in the cell [39]. The mechanism of dual targeting is driven by the competition of various molecular events. Protein folding, post-translational modification and protein-protein interaction are key players in this phenomenon [39]. Since proteins are generally considered to be translated in the cytosol, cytosol may be considered the default compartment for soluble proteins that do not transit to their initial programmed destination. The p49-N region contains three NLS motifs. Our data revealed that NDUFAB1 selectively interacted with p49-N region. It is possible that interaction of p49 with NDUFAB1 and other p49-binding protein(s) could make the NLS motifs of p49 protein unaccessible, thereby leading to the retention of p49 in the cytoplasm, as evidenced by the presence of p49 protein at low levels in the cytoplasm. Likewise, similar mechanisms could lead to the retention of NDUFAB1 in cytoplasm. It is also possible that the interaction between p49 and NDUFAB1 could retain both proteins within the cytoplasm, thus affecting their arrival at their programmed destination.

A recent study by Lisinski et al. reported that p49/STRAP is a cytosolic protein and was mainly localized to the cytoplasm [40]. Apparently Lisinski and colleagues performed microscopic analysis on the cells three days after transfection assay. In the current study, the cells were analyzed at 24 hours, 48 hours and 72 hours after the transfection was initiated. p49/STRAP protein was observed in the nucleus in all of cells in which transfection was successful. However, p49 was present in both nucleus and cytoplasm in a minority of the cells. In addition, the fraction of total p49 protein accounted for by the cytosolic portion seemed to decrease, both in absolute value and variability, with the degree of confluency. Therefore, the differences between the two groups could be partly due to different culture conditions. It is also possible that the p49/STRAP protein could have differing localization under different conditions. Protein modifications including phosphorylation may also influence nuclear transport of p49/STRAP.

Inasmuch as p49/STRAP interacted with NDUFAB1 and had an impact on the intracellular NAD/NADH ratio, it is possible that the interaction affects the assembly of Complex I. Recently, it was reported that the transcription factor TFII-I plays a role outside the nucleus as an inhibitor of the cell surface Ca^2+ ^channel TRPC3 by competing for binding to phospholipase C-g (PLC-g) [41]. Similarly, p49/STRAP may also play a role outside of the nucleus. It is also possible that binding of NDUFAB1 by p49/STRAP could alter the ability of NDUFAB1 to enter mitochondria and/or could affect the assembling of Complex I, thereby altering its function and possibly the NAD/NADH ratio.

In this study, it was found that overexpression of p49/STRAP reduced the level of acetylated-SRF protein, suggesting that p49/STRAP may play a role in the regulation of SRF through the process of acetylation and deacetylation. Recent studies reveal that reversible acetylation of non-histone proteins, including transcription factors, play an important role in the regulation of gene expression. The reversible acetylation of non-histone proteins is usually carried out by histone acetyltransferases (HATs) and histone deacetylases (HDACs). The HATs and HDACs are often found to be binding proteins of a number of transcription factors and cofactors, and have also been found to be in protein complexes containing certain transcription factors and cofactors [42,43]. In addition, certain transcription cofactors have been proven to be new members of a growing family of acetyltransferases and deacetylases)[42-45]. It will be interesting to know the mechanisms by which p49/STRAP induces the deacetylation of the SRF protein. It is possible that p49/STRAP may function as a deacetylase that deacetylates SRF directly. It is also possible that p49/TRAP change the intracellular NAD/NADH ratio thereby activating (or recruiting) certain deacetylase(s) which in turn reduce the level of acetylated-SRF protein.

## Conclusion

The findings in the present study demonstrate that p49/STRAP regulates intracellular NADH/NAD ratio and SRF deacetylation. The mechanism of regulation of NADH/NAD ratio by p49/STRAP is incompletely established at this time and remains to be further investigated. We plan to study the mechanism(s) in the future.

## Abbreviations

NADH: Nicotinamide adenine dinucleotide hydride; NAD: Nicotinamide adenine dinucleotide; p49/STRAP: "SRF-dependent transcription regulation-associated protein" which is a 49-kilodalton protein; SRFBP1: "serum response factor binding protein 1" which is a synonym of p49/STRAP; NDUFAB1: NADH dehydrogenase (ubiquinone) 1, alpha/beta subcomplex, 1, 8 kDa; HA: Hemagglutinin; SRF: Serum response factor; Zipzap/p200: "Zinc finger protein with activation potential" which is 200-kilodalton protein; EGFP: Enhanced green fluorescent protein; MOI: Multiplicity of infection; NLS: Nuclear localization sequence; pat4: Pattern-4; pat7: Pattern-7.

## Authors' contributions

Authors' contributions: XMZ participated in the design of the study, carried out most of the experiments and drafted the manuscript. GA participated in the coordination of the study and the writing of the manuscript. SH participated in the bioinformatics analysis and the intracellular localization studies. YZ participated in the yeast two-hybrid screening, production of recombinant adenoviruses, cloning and generation of constructs, cell transfection and immunoprecipitation. JYW led the study and participated in the design of the study, the statistical analysis and overall interpretation of results. All authors read and approved the final manuscript.
